# Sheep in the Vineyard: Suitability of Different Breeds and Potential Breeding Objectives †

**DOI:** 10.3390/ani12192575

**Published:** 2022-09-27

**Authors:** Lucas Conrad, Jakob Hörl, Maverick Henke, Rainer Luick, Nicolas Schoof

**Affiliations:** 1 Chair of Nature Conservation and Landscape Ecology, University of Freiburg, 79106 Freiburg, Germany; 2 Chair of Nature Conservation and Landscape Ecology, University of Applied Forest Sciences Rottenburg, 72108 Rottenburg, Germany; 3 Chair of Site Classification and Vegetation Sciences, University of Freiburg, 79106 Freiburg, Germany

**Keywords:** sheep breeding, viticulture, sustainable intensification, integrated crop-livestock system, ICLS

## Abstract

**Simple Summary:**

There is a large number of sheep breeds worldwide. Today, many of them are endangered, because there are very little market demands for the majority of breeds. The integration of sheep in viticultural systems offers a promising option to put sheep and unique breed characteristics in value. However, it is unknown which characteristics and breeds are best suited for this purpose. The lack of information leads to problems in the implementation of this so-called new integrated crop-livestock system. In our research, we addressed this challenge. We studied 26 breeds and tested their suitability for integration into common vineyards of Central Europe. Two breeds fulfill the most important requirement. Southdown and Shropshire, for the latter especially, the sheep of the shorter legged Danish type seem to be unable to stand on two legs. Their muzzle heights stay within a tolerable range without harming the foliage area of vines. Therefore, adult animals of both breeds seem suitable to take over important viticultural tasks during the growing season. A third breed, the Ouessant sheep, is suitable with some limitations.

**Abstract:**

Protecting a breed of sheep is simple when there is demand for its breed traits, but new market options are often hard to find. In general, grazing sheep are able to take over some viticultural work. Here, we address a new and promising integrated crop-livestock system that involves the integration of sheep in the vineyard during the growing season. Using sheep in a vineyard entails opportunities but also risks, such as the current lack of information, specifically in relation to breed traits. In our survey, we evaluated 26 breeds for their suitability for grazing as long as possible in Central European vineyards during the growing season. First, the breed traits required were identified. Then, 94 flock book breeders were interviewed about specific breed traits. The height of a sheep’s muzzle is particularly important for assessing the suitability of a breed, as it defines the potential impact on the foliage area during the growing season. To determine the height of the muzzle, 179 flock book animals were measured. We found that the most important breeding objective for a new breed of sheep is the inability to stand on two legs. Adult animals of the breed Shropshire, and among these especially the shorter-legged Danish type, and Southdown, show a widespread inability to stand on two legs. Ouessant sheep are able to do so, yet are suitable with some limitations. Due to their extraordinarily small size, their reach is limited, as is their grazing performance. Thus, three of the 26 breeds studied here seem suitable for use in the most widespread vine training systems of Central Europe during the growing season. Targeted breeding could further improve the suitability of sheep for viticulture. Our findings could help to protect breeds and breed traits.

## 1. Introduction

Worldwide, more and more livestock breeds are becoming extinct. This can be attributed to one decisive cause: demand for specific breed traits is declining in globalized agricultural markets and in modern agriculture. With this, options for adaptation to rapidly changing environmental conditions and land use are lost. The best solution would be to generate a corresponding demand for more livestock breeds; however, this is not easy [1,2,3]. The use of sheep to graze in vineyards during the growing season is a new form of integrated crop-livestock system (ICLS; [4]) that has hitherto scarcely been explored [5]. It has interesting potential in terms of agro-ecological system services [6,7,8,9,10], and it seems possible that specific requirements for breed traits could help to expand the market for sheep breeds or rare breed traits.

A recent survey of German- and French-speaking winegrowers who deploy sheep in their vineyards identified positive experiences, as well as limitations and problems of sheep deployment. Potential benefits and opportunities include: (1) higher land use efficiency through additional agricultural production (meat, wool); (2) reduction of external inputs; (3) stronger orientation towards nutrient cycles including potentially positive effects on ecosystem services; and (4) image enhancement for the winegrower with new marketing options [10,11]. Whether and to what extent these presumed potentials are activated surely depends on various influencing factors. Such factors are, for example, the duration of grazing, the grazing period, the accompanying vegetation or a possible additional tillage [12,13].

**Figure 1:**
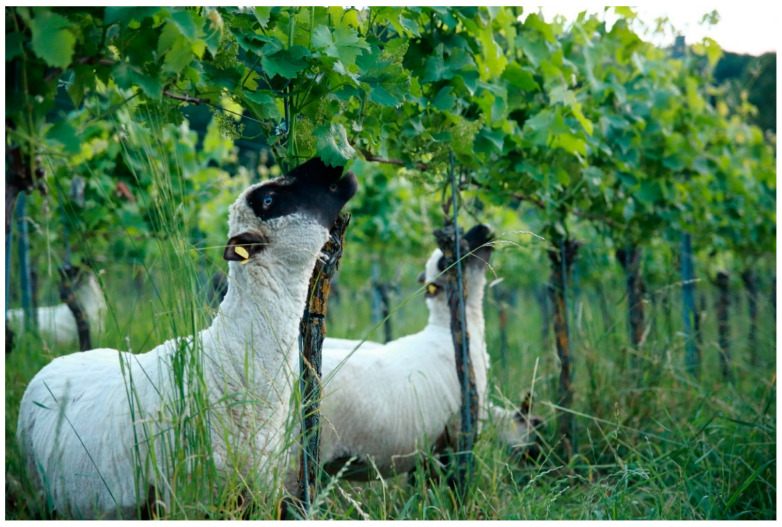
Shropshire sheep clearing the grape zone (leaf plucking) in the widely used Guyot vine training system. Here, a trellis (espalier) system is used to anchor the foliage area. The lowest wire is decisive for the height of the grape zone.

Sheep can support or partially replace certain labor-intensive viticultural tasks. These include: (1) weed management, including in-row floor management; (2) deterring wild animals that are problematic from a viticultural point of view (e.g., deer, rabbits); (3) removal of sucker shoots; and (4) removal of undesirable leaves from the grape zone to ensure grape and wine quality (called leaf plucking, Figure 1). This work is otherwise carried out mechanically, chemically or manually. The risks and disadvantages of deploying sheep in the vineyard arise: (1) from the time required to care for the animals; (2) from a necessary change in operational processes, which must necessarily be more oriented to the behavior of the animals; and (3) from the lack of information available hitherto. The latter encompasses, amongst others, veterinary issues and the lack of predictability regarding pasture management and its correlative effects on the foliage area of the vines. For example, research has yet to determine the height which different sheep breeds can reach when grazing and the minimum or optimal height at which the top of vine stems and leading- or fruit shoots should be located [10,11]. Niles et al. [6] show comparable results in a study conducted in New Zealand. The authors emphasize the positive business potential, which Jackson [5] describes more critically in his study. The different evaluation seems to be mainly conditioned by different implementation forms of the new ICLS. A further issue is that sheep in viticulture seem to require specific animal breed traits, which have to date rarely been relevant in breeding [10].

While today grazing with sheep can be observed more frequently in the winegrowing areas outside the growing season [9,12], the success of grazing during the growing season relies on various conditions and requires relevant know-how. However, there is currently a high level of interest in this model of land use. An important finding is that in the Guyot vine training system (Figure 1), which is the predominant form of vine training system in Central Europe, the possibilities of using sheep are mostly determined by the choice of breed. In contrast, vine training systems with spur pruning, minimal pruning or pergola training tend to give more options in breed selection. These training systems are quite rare in Central Europe and even here certain breeds are more suitable than others [10,11,14].

Sheep breeding was historically oriented to the respective regionally varying locational and economic requirements. Breeding resulted in significant phenotypic differences between breeds. We are not aware of any case in which breeding has been specifically targeted for use in modern permanent crop.

Since many countries do not keep lists of their autochthonous breeds, it can only be estimated how many sheep breeds there are worldwide. According to an overview by Oklahoma State University, about 200 breeds can be clearly distinguished [15]. A German association for ancient breeds estimates that there are about 600 breeds worldwide [16], and some sources estimate that there may even be 1000 breeds globally [17].

The low to non-existent economic value of wool and the existing need for annual shearing have encouraged breeding efforts towards natural wool shedding [18]. Breed-specific traits are also present in behavior. For example, Shropshire sheep do not browse conifers and can therefore be used in Christmas tree plantations for weed management. This aversion to conifers is not known to occur in any other breed. Breeding not only serves the conservation of existing sheep breeds; new breeding objectives can help to open up new paths for sheep deployment [6]. Furthermore, even within a breed it is possible to categorize different types that show distinct expressions. This is again the case for the Shropshire breed, where historically a Danish type has emerged with significantly shorter legs compared to the original breeding population (here the latter is simply called English type; recently, the types are becoming more and more interbred in the flocks of many hobby- and livestock farmers).

**Figure 2:**
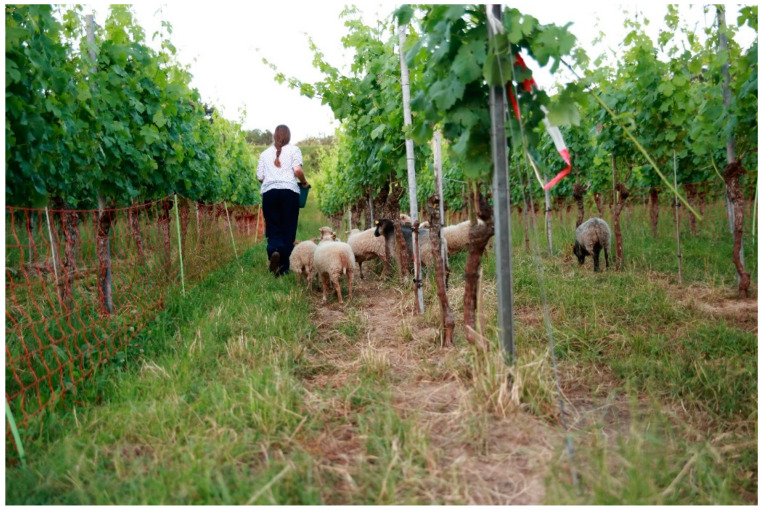
Ouessant sheep grazing in a vineyard with Guyot pruning (see above). In this viticultural system the browsing-sensitive (critical) phases of the growing season comprise the time from budding till the time berries reach groat-size and then from the veraison (start of the ripening of the grapes) through to harvest time. To avoid damage, sheep should not be allowed to graze vineyards during these critical phases. For grazing outside of the two described critical phases, some breeds seem much more suitable than others [10].

In view of the differences in breeds and breeding objectives described above, it is hardly surprising that the suitability of breeds for use in viticulture should be assessed in a differentiated way. The following example shows that sheep breeds display very pronounced differences: ewes of the smallest sheep breed in the world, the Ouessant (Figure 2), weigh only 13–16 kg [19]; while ewes of the breed Berrichon du Cher can reach up to 100 kg [20]. The question of the suitability of specific breeds applies in particular to the widely used Guyot vine training system. The leaves of the vine foliage are often at or below the height reachable by the muzzle of many breeds of sheep. This issue is especially relevant when the animals are able to rise up on their hind legs to reach higher leaves after having eaten the lower ones. The German pioneers of the use of sheep in the vineyard mainly deploy the Ouessant breed, sometimes called dwarf sheep. However, larger breeds such as German Heaths, Shropshire, Cameroon or Suffolk are also used in individual cases [10,11].

In order to minimize existing viticultural risks in the deployment of sheep and to exploit potentials, a sheep breed should meet criteria that explicitly correspond to viticultural requirements. First, it is necessary to identify these requirements and second, to analyze breed-typical traits for their suitability [21]. In this way, suitable breeds (or types of within breeds) can be distinguished from less suitable ones. In addition, a breeding objective for husbandry or parameters for breeding new (not yet existing) sheep breeds can be defined. Identifying these requirements, including the suitability assessment of different sheep breeds, was the research objective of the present study. The study is semi-quantitative. Its aim was not to make definitive statements, but to give valid recommendations, which will have to be further verified in the complex systems of application.

The questions we pursued in our study were:

1. Are there breed characteristics that are necessary or desirable for grazing Central European viticultural systems during the growing season? If so, what are they?
2. Which breeds would be most suitable on the basis of the recommendations made in answer to 1?

## 2. Materials and Methods

Due to the variety of grapevine training forms and forms of sheep husbandry, numerous ways of applying the ICLS “sheep in the vineyard” are conceivable. It was therefore necessary to narrow down the parameters for the research objective. The focus of the search for (particularly) suitable sheep breeds and traits is on their suitability for grazing as long as possible during the growing season, and here specifically on the predictability of the effects regarding the amount of defoliation of plants in the Guyot vine training system (first wire defining the grape zone at ~90–105 cm). In most cases this premise also covers the suitability for use in minimal pruning and top-wire cordon vine training systems. In the latter, grazing is less of a challenge, because the grape zone is higher. In practice, a potential deployment of sheep is limited by: (1) the developmental stages of the vine (sheep only avoid berries with a certain acidity); (2) the use of plant protection products; and (3) the details of the vine training (= height of the cane, i.e., the lowest wire, as well as the height of the vine stem) [10].

At a meeting in December 2019 with pioneers of the new ICLS, as well as interested winemakers (31 participants), viticulturally desirable sheep breed characteristics were first identified. We used expert-interviews and discussions here [22]. Based on these findings, a literature- and expert-supported pre-selection of potentially suitable breeds was made, taking into account their actual availability in Germany. In order to supplement or verify the spectrum of sheep breeds that could principally be considered, the results of the only scientific study available to date for Central Europe on the implementation of the ICLS were used. This study identifies sheep breeds that are already used in vineyards in Central Europe [11]. 

The surveys conducted for the study were explicitly for adult females, as they are in principle more suitable than rams or castrates for vineyards. Females have the following advantages: (1) they can be used for offspring; (2) males cause additional costs due to the potential need for castration; and (3) in most sheep breeds, females are hornless, which simplifies management and control [23]. In practice, sexually mature rams will probably only be present individually or of small numbers in a flock, although ram flocks in the vineyard are also conceivable in principle.

Initially, 26 breeds were included in the pre-selection. The multipurpose breeds include Alpine Stone Sheep, Braunes Haarschaf *, Cikta Sheep, German Grey Heath, Herdwick, Bovec Sheep, Montafon Stone Sheep, Ouessant, Pomeranian Coarsewool Sheep, Scottish Blackface, Skudde, Soay Sheep *, Racka, Waldschaf, Wallachian, Roux du Valais, White Horned Heath and White Polled Heath. For meat sheep breeds, they were Barbados Blackbelly*, Berrichon du Cher, Charmoise, Charollaise, Dorper, Cameroon Sheep *, Olde English “Babydoll” Southdown, Shropshire (English and Danish type), and Southdown (breeds marked with * are hair sheep breeds [11]).

Breed-specific characteristics that are explicitly interesting for use in the Guyot vine training system are not always mentioned in the existing breeding specifications and are partly undocumented in the literature. Flock book breeding, for example, provides specifications for a breed-specific permissible (size) range of wither height. However, it is the height of the muzzle that is decisive for viticulture. Though this normally correlates anatomically with the wither, the exact correlation is unknown. In addition, flock book breeding does not provide any guidelines for bipedal ability or inability, which many breeds are demonstrably capable of, contrary to descriptions [24]. The ability to stand bipedally is crucial for the absolute grazing height and the predictability of the sheep’s effect on the vine foliage area and thus influences the potential grazing time. The risk of undesirably high defoliation or damage to shoots and panicles increases with bipedal ability and the correspondingly raised height of the muzzle [10].

**Figure 3:**
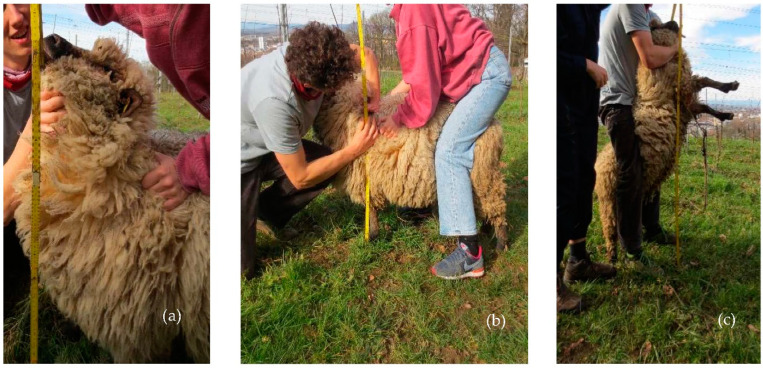
Exemplary measurement of: (**a**) max. grazing height in four-legged stance; (**b**) wither height; and (**c**) max. grazing height in two-legged stance of a Shropshire.

For a suitability assessment, therefore, these desired breed traits first had to be recorded. For our survey, contacts with flock book breeders were established. Flock book breeders subject their breeding to defined breeding standards. Their sheep are therefore representative for the breed. Contacts were identified via the “*Schäfereikalender 2020*”, an address book of the German breeding associations [25], or via websites of the regional sheep breeding associations [26]. Data on the relevant breed traits were collected using two methods:

1. Data collection on the animal: measurements of the height of the sheep’s muzzle from the ground, which is particularly relevant for viticulture (for explanation see also results section), were in each case carried out with the respective breeds standing still on level ground. Wither height, the height of the muzzle with stretched neck and the head pointing upwards, as well as the maximum height of the muzzle in two-legged stance (if the breed is capable of this—see below) were recorded. For the latter, sheep were manually lifted onto their hind legs. An attempt was always made to imitate the natural posture of the sheep (exemplarily Figure 3). Since this is not absolutely possible, a “range of variation” should be considered in practice. Based on the trials, +/− 10 cm was defined as a potential variation for the maximum grazing height. Measurements were carried out at 25 farms on a total of 19 sheep breeds and 179 sheep. The number of sheep measured per breed was dictated by the current availability and varied between four and 20 sheep. We continued to distinguish within the Shropshire breed between the Danish and English type, since the anatomical differences in terms of leg length can be large. We assumed that this could affect the ability to stand bipedally (Table 1). For the following pre-selected breeds, no sheep could be obtained: Cikta, Montafoner Stone, Scottish Blackface, Soay, Recka, Wallachian and Roux du Valais.
2. In addition to these measurements, robustness, manageability and bipedal ability were recorded as other relevant characteristics via standardized questions to the breeders. A total of 94 breeders were interviewed. The interviews were recorded. For 23 breeders the interview was conducted on site, for 71 breeders, by telephone. The question regarding robustness asked specifically about: (1) resistance to weather conditions; (2) hoof constitution; and (3) fertility. The positive answers were marked with “+”. For the Cikta sheep and the Montafon Stone sheep, no surveys could be conducted (Table 2).

In order to estimate the expected grazing performance (time in which an area is grazed) as a potential breeding or suitability characteristic, a breed-specific calculation of livestock units (LU) was carried out. In Appendix II, Article 9 (1), (2) of the EU’s Commission Implementing Regulation No. 2016/669, 0.15 LU are used for one sheep. Assuming that this is a female Merino (the most common breed in Central Europe) with an assumed weight of 85 kg, a specific LU for each breed can be derived. For derivation, the mean value of the weight range tolerated in breeding for the respective breed was correlated with the above-mentioned EU estimate.

**Figure 4:**
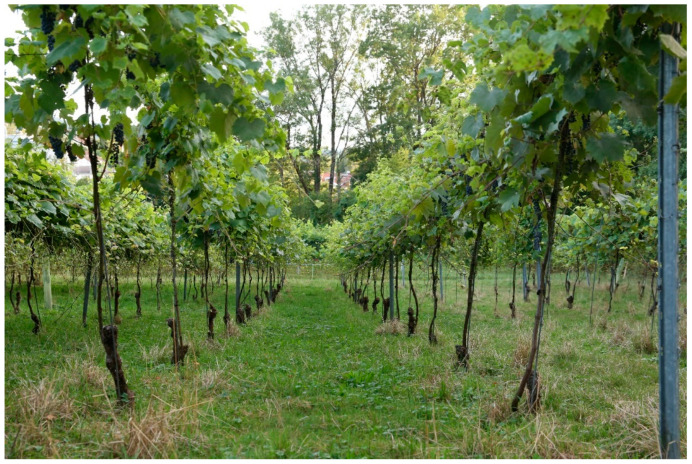
Top-wire cordon system. This vine training system is rather uncommon in Central Europe. It would be optimal for grazing because the grape zone is very high (>140 cm). In our study, we focused on a viticultural system that is more widespread. In this common system (see Figure 1 and Figure 2), the browsing-sensitive phases of the growing season last from several weeks to a few months, depending on the grape variety. In such phases, grazing, even with breeds that are suitable in principle, is only possible if the flowers and grapes are protected from browsing (e.g., with an electric wire).

A post-disciplinary research approach was used for the study [27]. The authors themselves run 30–50 animals of the breeds Shropshire (Danish and English type as well as their crossbreeds) and Ouessant. The animals are used during the growing season and non-growing season in vineyards with the Guyot system, and in minimal pruning and top wire cordon training systems (Figure 4). The vineyards are located in Freiburg, Germany. The experience acquired over three years helps to better describe, evaluate and classify the results of the study.

## 3. Results

### 3.1. Beneficial Phenotypic Traits

The participants of the expert workshop agreed that there are viticulturally desired breed traits. These are of particular importance for the new ICLS described above. Participants considered the following traits to be favorable (ranked in descending order of importance):

1. Bipedal ability: This feature limits the potential grazing height on the foliage area and therefore favors the potential deployment during the growing season in the Guyot system. For sheep without bipedal ability, the effect of grazing is more predictable and the components of the system (especially the height of the lowest wire and grazing time) can be adjusted with predictable results (nevertheless, there are two critical phases, Figure 2). In other vine training systems, this feature may be less important, but it is by no means insignificant [10]. A two-legged stand would be only tolerable if the muzzle of the sheep remains sufficiently low.
2. Robustness: This factor reduces demands on the know-how of the owner, the amount of work and potential veterinary costs, and is generally beneficial to animal welfare. It is therefore a common breeding objective for all breeds. In addition, the animals should be as insensitive as possible to pesticides applied in vineyards. For example, copper, which is used specifically in organic farming, brings a potential risk of chronic poisoning to sheep. Copper sensitivity is breed-specific, however, within certain limits [28]. This last requirement was not pursued here, but initial research findings are available on the topic [9]—the toxicity of other pesticides is insufficiently known in many cases.
3. Manageability: Docile, manageable animals simplify the work during a change of pasture when a sheepdog cannot be used. In vineyards where the winegrower is also the shepherd, the sheep’s docility is more important than in the case of professional shepherds. The latter usually have herding dogs [29], which winegrowers tend not to keep. Moving the animals without a herding dog is more or less easy depending on the breed.
4. Natural wool-shedding: The need to save time and labor is the reason for dispensing with sheep-shearing. In any case, the cost of shearing today often exceeds the proceeds from the sale of wool [18].
5. Grazing performance: To be determined approximately via the average weight of a breed according to breeding specifications. Axiom: the higher the weight, the higher the grazing performance, the fewer animals are needed for a comparable area. Fewer animals, in turn, correlate with savings in labor, time and monetary costs for animal management.

The participants of the event unanimously confirmed the above-listed points. The participants emphasized that, depending on the farm and specific viticultural training system in use, the importance of individual characteristics can of course be more or less important. For example, manageability is less important for a farm that cooperates with a shepherd who keeps sheepdogs. In the discussion it was noted that the list and the weighting reflect the current status for the new ICLS described above. It could change, for example, if viticultural training systems change widely.

### 3.2. Results of Measurements and Survey of Breeders

**Figure 5:**
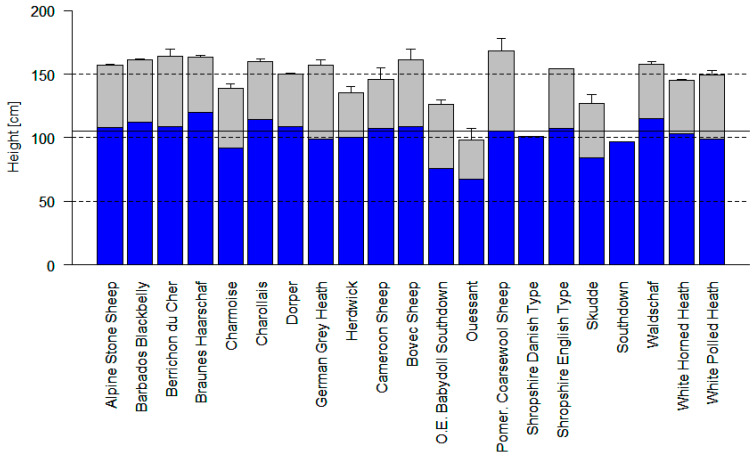
The muzzle heights of the sheep breeds studied. The dark blue area shows the average height of the muzzle, which is reached in the four-legged stance with the neck stretched. The gray area shows the range of heights that can additionally be reached in the average of the measurements, given the ability to stand bipedally. The whiskers show the maxima of the measurements (for number see Table 3). The solid line at 105 cm is the height of the lowest wire, which was proved to be favorable for deployment of the Shropshire Danish type in our own field trial (several weeks of deployment in the Guyot system during the vegetation period is possible). The lower limit is 105 cm (see below). Within the English type of Shropshire, there appear to be many suitable animals that are not capable of standing bipedally. O.E. = Olde English; Pomer. = Pomerian.

Two of the sheep breeds studied (Southdown and Shropshire) are, according to breeders, predominantly incapable of standing bipedally. However, in the case of the Shropshire breed, this seems to apply more to the compact and shorter-legged Danish type. Within the English type, especially when of flock book breed origin, there is a higher chance that some individuals may be capable of a two-legged stance. Ouessants are predominantly capable of such a stance, but the breed is much smaller than the other breeds studied; even when standing on two legs, the muzzle height of the Ouessants remains below that of other breeds in the four-legged stance (Figure 5). Ouessants are therefore suitable in principle for use in the Guyot vine training system.

The LU range across all breeds is between 0.026 (Ouessant) and 0.172 (Charollais). In order to achieve the grazing performance of a Charollais ewe, 6.6 Ouessant ewes would have to be used. All of the breeds are considered by flock book breeders to be robust in terms of their resistance to the effects of weather, their hoof constitution and their fertility. Ten breeds were positively evaluated for their manageability, 13 were negatively evaluated by flock book breeders, and two received conflicting responses from breeders (Table 3).

## 4. Discussion

It should be emphasized that our study was a first qualitative screening for the suitability of breeds for the new ICLS. First of all, we were able to identify breed characteristics that can be considered favorable for grazing common vineyards of Central Europe during the vegetation period. The list of these traits could change, for example, if current viticultural systems are strongly adapted in the future. However, we consider the suitability list of breed traits to be efficient and effective for other viticultural practices as well, so we do not expect any significant changes to occur. In our own herd, we could observe that even in minimal pruning systems, much better results are obtained when the animals cannot stand on two legs. The inability of standing bipedally is a key requirement for long-duration grazing during the growing season.

Breed-specific differences in the ability to stand bipedally have already been documented for goat breeds [30]. To our knowledge this has never been systematically recorded for sheep. For some breeds, only a limited number of animals could be measured and/or a small number of breeders could be involved in our study. Assuming there is a significant correlation between breed-specific breeding requirements (especially with regard to wither height) and the height of the muzzle in the four-legged or bipedal stance, measurements of a few animals give a good but not final indication of the suitability of a breed for the integration into the targeted vine training system (see above) during the grazing period. In our study, the in some cases small number of measurements did not matter significantly, as breeders of the affected breeds confirmed that sheep of their breed are capable of standing bipedally. Specifically, it would have been desirable to involve more breeders for the evaluation of Southdown, because this breed seems to be suitable for the new ICLS. However, this breed is relatively rare in German-speaking countries. Therefore, we could not identify any further breeders, so that our recommendations for Southdown need to be interpreted with greater caution. 

**Figure 6:**
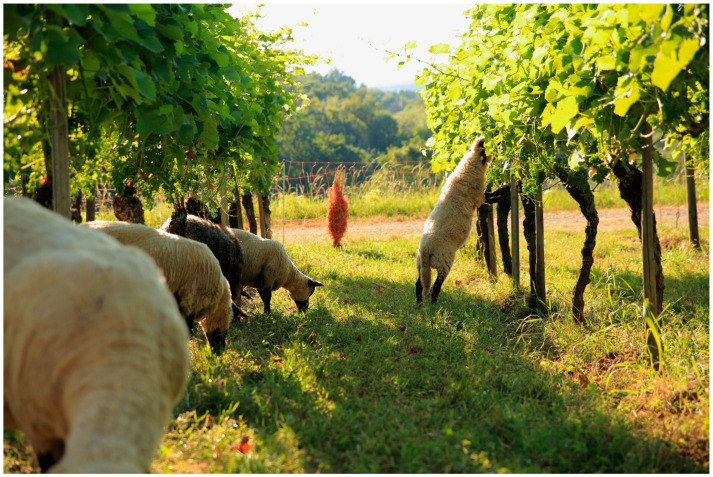
Sheep in bipedal stance. There are breeds suitable for grazing for several weeks during the growing season in common viticultural systems of Central Europe. Many breeds and animals are only suitable to a limited extent because they are capable of bipedal grazing, as seen here in the photo.

Southdown, Ouessant and Shropshire are, according to our survey, much better suited than the other 23 breeds evaluated for the longest possible deployment in the vineyard during growing season. Either they are very small (Ouessant) or are predominantly incapable of a bipedal stance. In Shropshire of the Danish type, problems with lambing appear to be relatively widespread. To remedy this and to improve the growth, both types are often intercrossed. Within our own Shropshire flock, some animals have parents of both types and are incapability of a bipedal stance. The relationship of body weight to leg length seems to be decisive, while relatively longer legs tend to correlate with bipedal ability. Yet, our data does not allow a conclusive assessment on this point. In contrast to the Ouessant (0.026 LU), Shropshire and Southdown also have more favorable characteristics in terms of manageability and grazing performance (Shropshire: 0.136 LU; Southdown: 0.127 LU), so the time, labor and monetary costs of husbandry will be less than with those of Ouessant. The breeds identified in our study can potentially also be utilized for deliberately (further) breeding a “vineyard sheep breed”. Only a few of the breeds studied seem suitable for crossbreeding in the sense of producing such a new breed (Figure 6). In order to breed-in naturally fleece shedding qualities to Southdown or Shropshire, Dorper in particular seem to be promising. Although natural fleece shedding saves labor and cost of shearing, from the point of view of land-use efficiency this feature would be regrettable, even if the wool prices in Central Europe currently favor naturally fleece-shedding breeds. 

It is worth to mention, that in many cases, flock book breeders exclude animals that do not meet breeding standards, but whose phenological characteristics might be particularly suitable for the use in vineyards. These individuals can only be identified through direct contact with sheep farmers and breeders. Based on our own experience, within the Cameroon breed there are small-sized rams with a wither height of less than 60 cm. Such animals do not appear in flock books and were therefore not included by the selected method. For instance, with these types of individuals, the trait of natural wool-shedding could possibly be crossed-in in Ouessants. Ouessant rams could also be crossed with (the larger) Cameroon. However, even with flock book breeding, not all individuals are comparable in terms of behavior [31,32]. Based on our observations, not all individual Ouessant sheep, for example, make use of the two-legged stance. Such behavioral differences in individuals are not documented by the flock book breeders. It is possible that even flock book breeders who have sheep which are in principle capable of standing bipedally, would keep individuals that are unable or unwilling to do so and would therefore potentially be well suited for the breeding of vineyard sheep. The reverse case is also conceivable; in this case, animals capable of bipedal stance would have to be excluded. For example, within the English type of Shropshire, there appear to be many suitable animals that are incapable of standing bipedally. According to our observations, leg length seems to be a good predictor of this ability, and would serve as an ever better predictor if the differentiation between English and Danish type were omitted. It is also worth adding that lambs may exhibit again aberrant behavior in comparison to adults [11], which would need to be taken into account in viticulture. We were unable to consider non-adult animals in our study. Nevertheless, it is clear that a flock deployed in the vineyard during the growing season must, if possible, be further bred to produce or retain the desired traits.

The breeds Ouessant, Shropshire and Southdown thus predominantly fulfill the identified priority traits for better predictability of grazing on the foliage area in a widely used vineyard training system and for minimizing the risk of undesirable grazing effects. This is true, however, with the following caveat: both the height of the lowest training wire of the vine training system and the height of the vine stem varies in different vineyards, so that the suitability cannot be seen in absolute terms, but only as a function of the design of the espalier. For example, if the grape zone is at 70 cm (which is in some vineyards the case), the deployment of sheep described above is virtually impossible across all breeds during the growing season. 

In the course of climate change and increasing global warming, creating a shorter foliage canopy by raising the lowest wire is fairly unproblematic from a viticultural point of view, and often even advisable. Adjusting the wire frame in training systems is surely not always possible. In addition, the above-mentioned range of variation (+/−5 cm in four-legged stance) must be taken into account when predicting the grazing height. In our own trials, we obtained some excellent leaf plucking results, but the predictability of effects has limits and also requires a change in thinking away from the more precisely predictable results of using machines for the same task.

**Figure 7:**
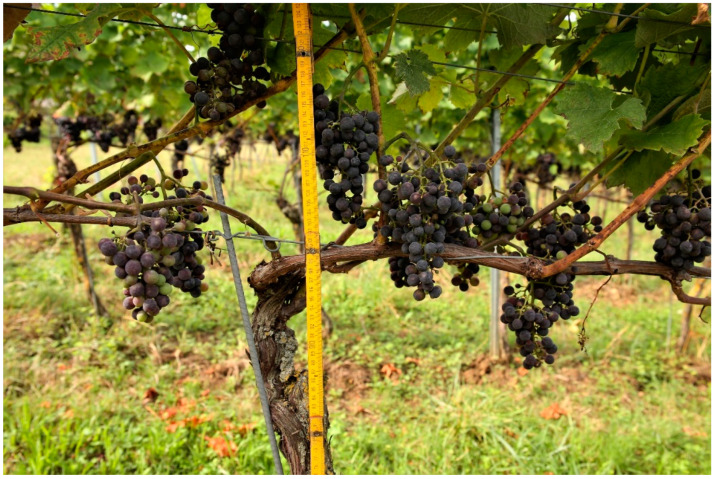
Results after several weeks of grazing and leaf plucking with a small Danish-type Shropshire flock. The result was considered favorable by the winemakers. Optimization could be expected if the lowest wires were raised a little more. Alternatively, the animals would have to be removed from the pasture earlier. As an approximation, the following guideline is valid: the longer sheep are in the vineyard, the higher they defoliate. Breeds that are capable of bipedalism start to use it over time.

**Figure 8:**
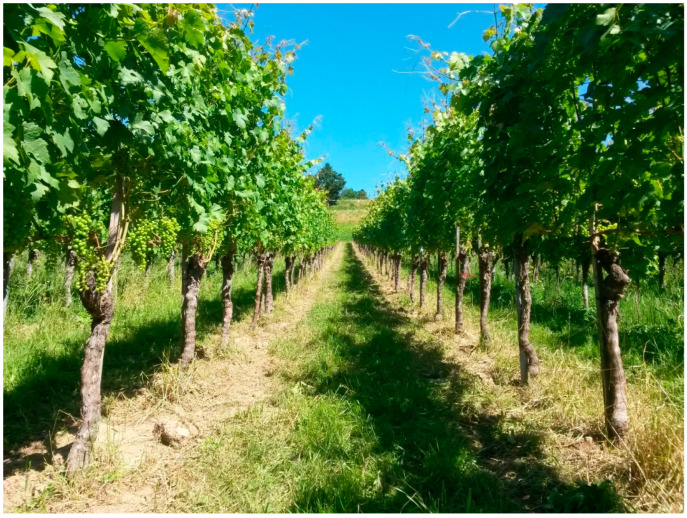
Effects after four days of grazing on approximately 450 m^2^ of vineyard with 20 Ouessant sheep. To be seen here: inter-row vegetation grazing incl. in-row weed management (here also due to scratching and lying down), almost complete and highly welcome removal of sucker shoots as well as nearly perfect grape zone clearance (leaf plucking). Ideally, animals should change surfaces when the desired effect is achieved. Animals could possibly be forced by the lack of accompanying vegetation to make stronger efforts to reach higher vine leaves or to eat unripe grapes. Only those breeds whose wither heights are below the wire frame and any applied irrigation systems (so that these are passable) are likely to be suitable for short-term growing season deployment. If grazing is to be carried out over a longer period of time (several weeks) in Guyot systems, however, the majority of the evaluated breeds must either be discouraged or a relatively large number of adaptations to the vineyard must be made.

From which height of vine stem and first wire sheep can be deployed or deployed in an optimal way is a question of the intended/used sheep breed. Using the present findings, grapevine training can now be adapted to the muzzle height of the suitable breeds in order to approach the optimum. For the previously mentioned parameters (height of first wire, fruit/leading shoot, height of vine stem), fully validated, application-related information is still lacking, but in our own practical trial, a height of 105 to 115 cm (depending on the size of the individual sheep: we used the Danish and English type) for the lowest wire/cordon is a perfect height for the deployment of Shropshire (Figure 7). With fast grazing rotations, i.e., of only a few days, which focus exclusively on grape zone clearance, favorable results were also achieved where the lowest wire was between 90 and 100 cm high (Figure 8). This also applies to vines on steep slopes.

Due to their anatomy and strength, Southdown and Shropshire are, according to our experience, generally much more capable than Ouessant of pulling fruit shoots out of the espalier framework and thus causing damage (the stems dry out). In part, this behavior is learned only over time. For this reason, in addition to adapting the wire arrangement (see above), it seems advisable to use the highest possible vine stem to explicitly avoid this risk. The leverage that the sheep can achieve by pulling with its mouth is lower with high vine stems and the correspondingly higher base of the leading shoots. It is possible that the (more costly) cordon cut would be more favorable for sheep use, since the young shoots attach here at the level of the lowest wire. However, a good vine training system (insertion of the shoots into the espalier framework) is always a prerequisite for the use of sheep, provided that they can reach the fruiting shoots [11].

In summary, our study has shown that there are large differences in the characteristics of sheep breeds. Our findings indicate that there is no one breed among those studied, which has all the traits desirable in viticulture. This does not rule out that said mix of traits may be found amongst the multitude of breeds existing worldwide. Southdown and Shropshire (especially the Danish type) can already be attributed four of the five qualifying breed traits listed above. As can be seen, breeding in the individual flocks of sheep kept for grazing vineyards should always be focused on promoting viticultural desired traits. In addition to sheep breed selection, several options for managing the risks and opportunities of the new ICLS have been mentioned. For example, installing electrical wires in parallel and below the foliage area could partially eliminate the dependence on breed selection in Guyot-trained vineyards.

Gonçalves et al. [33] investigated the possibility of using high-tech on sheep in vineyards. Collars should set acoustic and electric impulses when sheep feed on leaves or grapes. This approach was intended to reduce the labor involved in livestock keeping and is intended to allow year-round grazing. We are critical of this approach. First of all, it must be stated that the browsing of vine leaves can be a viticulturally desired effect. Besides the hoped-for benefits of high-tech, it is expensive, requires additional technical know-how and promotes technology dependency. In the study of Gonçalves et al. the sheep did not behave as desired, despite the electric stimuli. The authors furthermore suggest that grazing during the growing season is not possible without such technology [33]. We demonstrated in a previous study that desired results of the new ICLS are quite possible without high-tech [10] (see Supplementary Materials at the end). In the present study, we have shown that grazing vineyards is much more likely to be successful and effective when conditions are matched. 

In sum, the results of the precent study make it possible to further promote the new ICLS in common vine training systems of Central Europe. Based on our findings, the options for implementing the new viticultural model are considerable and suitable breed traits are already available. Clearly, the new ICLS has the potential to protect the mentioned breeds and breed traits. The tasks of future research fit into an established field of research on the transformation of sustainability-deficient land use systems [34,35]. There are still some open questions in the implementation of the new ICLS. Applied research should focus on testing more breeds, other livestock, alternative viticultural systems designed to integrate livestock, veterinary aspects and agro-economic as well as agro-ecological impacts of the new ICLS.

## 5. Conclusions

We were able to identify breed traits that are very important for integrating sheep in commonly used vine training systems of Central Europe during the growing season. The anatomical inability to stand on two legs, in particular, is crucial. Presumably, this breed-specific trait has not yet been recognized as important for other land-use forms. The new ICLS could open up new options for breeds that show this and other suitable traits. Ideally, the use of sheep would be considered when new vineyards are planned. Existing vine training systems can also be adapted to some extent for better and safer integration of sheep. Nevertheless, the breeds and breed traits recommended in our survey provide an excellent starting point for spreading the new ICLS. Research and practical testing of this ICLS has only begun, but could be highly significant in transforming a sustainability-deficient viticulture.

## Figures and Tables

**Table 1:** Information on the procedure for determining the grazing height.

Breed	Number of Sites at Which Measurements Were Carried out	Number of Sheep Measured	Own Measurement or Measurement by Breeders?
Alpine Stone Sheep	1	15	Own
Barbados Blackbelly	1	5	Own
Berrichon du Cher	2	8	Both
Braunes Haarschaf	1	5	Own
Charmoise	1	5	Own
Charollais	1	8	Own
Dorper	1	7	Own
German Grey Heath	2	12	Own
Herdwick	1	8	Own
Cameroon Sheep	1	4	Breeder
Bovec Sheep	3	20	Own
Olde English “Babydoll” Southdown	1	5	Own
Ouessant	2	14	Own
Pomerian Coarsewool Sheep	2	10	Own
Shropshire, Danish type	2	5	Own
Shropshire, English type	3	11	Own
Skudde	1	11	Own
Southdown	2	12	Both
Waldschaf	1	6	Own
White Horned Heath	1	4	Own
White Polled Heath	1	4	Own

**Table 2:** The number of surveys of breeders conducted per sheep breed to identify breed-specific suitability for deployment in the vineyard in growing season.

Number of Surveys Conducted Among Breeders	Breed
none	Cikta Sheep, Montafoner Stone Sheep
1	Charmoise, Olde English “Babydoll” Southdown, Roux du Valais
2	Southdown
3	Herdwick, Racka
4	Barbados Blackbelly, Scottish Blackface
5	Alpine Stone, Berrichon du Cher, Braunes Haarschaf, Charollais, Dorper, German Grey Heath, Cameroon, Bovec, Ouessant, Pomeranian Coarsewool, Shropshire, Skudde, Soay, Wallachian, Waldschaf, White Horned, White Polled Heath

**Table 3:** Results of the survey of phenotypic traits.

Breed		N	Muzzle Height TLS [cm]			Muzzle Height FLS [cm]			Manageability	NWS	LU
			Min	Max	Mean	Min	Max	Mean			
Alpine Stone Sheep		15	155	158	157	104	110	108	+	No	0.095
Barbados Blackbelly		5	160	162	161	107	114	112	+	Yes	0.095
Berrichon du Cher		8	154	170	164	100	117	109	−	No	0.154
Braunes Haarschaf		5	161	165	163	118	121	120	+	Yes	0.127
Charmoise		6	135	142	139	88	96	92	−	No	0.1
Charollais		8	158	162	160	111	118	114	−	No	0.172
Ciktaschaf		N/A								No	0.086
Dorper		7	148	151	150	107	111	109	−	No	0.136
German Grey Heath		12	155	161	157	95	106	98	−	No	0.09
Herdwick		8	128	140	135	64	104	100	+	No	0.081
Cameroon Sheep		4	107	155	146	97	115	107	−	Yes	0.077
Bovec Sheep		20	107	170	161	104	120	109	+	No	0.09
Montafoner Steinschaf		N/A								No	0.081
Olde English „Babydoll“ Southdown		5	120	130	126	74	80	76	+	No	N/A
Ouessant		14	90	107	98	57	77	67	indifferent	No	0.026
Pomerian Coarsewool Sheep		10	160	178	168	98	120	105	+	No	0.109
Scottish Blackface		N/A							indifferent	No	0.095
Shropshire	Danish type	5	unable			95	109	106	+	No	0.136
	English type	16	154	154	154	102	109	107			
Skudde		11	119	134	127	79	90	84	−	No	0.063
Soayschaf		NA							−	Yes	0.045
Southdown		12	unable			90	106	97	+	No	0.127
Racka		N/A							−	No	0.077
Walachenschaf		N/A							−	No	0.087
Waldschaf		6	156	160	158	104	115	112	−	No	0.086
Roux du Valais		N/A							+	No	0.113
White Horned Heath		4	144	146	145	103	103	103	−	No	0.081
White Polled Heath		4	145	153	149	98	99	99	−	No	0.081

Robustness reflects the breeders’ assessment of resistance to weather conditions, hoof constitution and fertility. Mean and median differ by a maximum of 1 cm. Taking into account individual anatomical differences, an interpretation of the max. height of the muzzle must recognize additional height spans: for the two-legged stance approx. +/−10 cm and for the four-legged stance approx. +/−5 cm. LU = livestock unit of the average weight of an ewe (conversion of 85 kg is 0.15 LU); N = number of sheep measured; N/A = not available; NWS = natural wool shedding; TLS = two-legged stance; FLS = four-legged stance; + = positive; − = negative; robustness to disease was rated positively across all breeds.

## Data Availability

The data can be requested from the authors if interested.

## Supplementary Materials

Under the following link you can see a video that shows an implementation of the ICLS (German language): https://www.3sat.de/wissen/nano/201006-schafe-nano-102.html Note: Bird protection nets can be seen in the video. The sheep, especially lambs, have learned after about a year to bypass this protection by pressing their heads between the nets.
